# Community knowledge, attitude, and perceived stigma of leprosy amongst community members living in Dhanusha and Parsa districts of Southern Central Nepal

**DOI:** 10.1371/journal.pntd.0007075

**Published:** 2019-01-11

**Authors:** Rakesh Singh, Babita Singh, Sharika Mahato

**Affiliations:** 1 Patan Academy of Health Sciences, Lalitpur, Nepal; 2 National Medical College and Teaching Hospital, Tribhuvan University, Birgunj, Parsa, Nepal; 3 Adventist Development and Relief Agency Nepal, Kathmandu, Nepal; 4 The Hebrew University-Hadassah Braun School of Public Health and Community Medicine, Jerusalem, Israel; Federal University of Agriculture, NIGERIA

## Abstract

**Background:**

Though Nepal declared leprosy elimination in 2010, its burden is constantly rising in Terai communities for the past 2 years with 3000 new leprosy cases being diagnosed annually. Community’s perception is important for prevention and control of leprosy and enhancing quality of life of leprosy patients. Poor knowledge, unfavorable attitude and stigma create a hindrance to leprosy control. The main objective of this study was to assess the knowledge, attitude and stigma of leprosy amongst the community members living in Dhanusha and Parsa districts of Southern Central Nepal.

**Methods:**

A total of 423 individuals were interviewed using a structured questionnaire in Dhanusha and Parsa districts. Data was analyzed using both descriptive (frequency, percentage, median) and statistical inferences (Chi-square test, Kruskal Wallis H test, Mann Whitney U test, binary logistic regression) using SPSSvs20.

**Results:**

All respondents had heard about leprosy. Source of information on leprosy was mainly found to be health workers/hospitals (33.1%). Only 62.6% reported bacteria being its cause followed by other myths such as bad blood/curse/heredity/bad deeds (36%). Only 43.8% responded that leprosy is transmitted by prolonged close contact with leprosy patients and 25.7% reported religious rituals as the treatment. Only 42.1% had good knowledge and 40.9% had favorable attitude. Good knowledge of leprosy was highly associated with favorable attitude towards leprosy (P<0.001). The outcome variables- knowledge, attitude and EMIC score were found to have highly significant association with age, sex, ethnicity, religion, education and occupation of the respondents (P<0.001). Having knowledge on leprosy transmission was positively associated with favorable attitude towards leprosy (P<0.001).

**Conclusions:**

Strategizing the awareness programmes according to socio-demographic characteristics for enhancing the knowledge regarding leprosy cause, symptoms, transmission, prevention and treatment, can foster the positive community attitude towards leprosy affected persons. Enhancing positive attitude towards leprosy affected persons can reduce the community stigma, thus may increase their participation in the community. Positive attitude may further increase their early health seeking behaviour including their quality of life.

## Author summary

Though Nepal declared leprosy to be no more a public health problem in 2010, its burden is constantly rising in Terai communities for the past 2 years with 3000 new leprosy cases being identified annually. With the fact that community’s knowledge and perception is important for prevention and control of leprosy this study aimed at assessing the community knowledge, attitude and stigma of leprosy amongst the community members living in Dhanusha and Parsa districts of Southern Central Nepal. The study was conducted in the communities of Dhanusha and Parsa by interviewing 423 individuals using structured questionnaire. All study respondents had heard about leprosy with main source of information to be health workers/hospital. A good proportion had myths such as bad blood/curse/heredity/bad deeds as the cause of leprosy and reported religious rituals as its treatment. Although more than half had good knowledge, only 2/5^th^ had favorable attitude. The attitude was found to be influenced by knowledge. Also, knowledge, attitude and stigma score were found to be influenced by age, sex, ethnicity, religion, education and occupation. Strategizing the awareness programmes according to socio-demographic characteristics for enhancing the knowledge regarding leprosy cause, symptoms, transmission, prevention and treatment, could change the attitude to make it more favorable and thereby would help in reducing leprosy burden and enhancing the quality of life of leprosy patients.

## Introduction

Leprosy, also known as Hansen’s disease, is a chronic infectious disease caused by bacteria *Mycobacterium leprae* [1]. It generally affects epidermis and peripheral nerves of the affected ones [2]. The disease is basically transmitted via prolonged close contact with untreated multibacillary leprosy patients through inhalation of bacilli [2; 3]. However, it is still an unequivocal issue regarding transmission of leprosy from one person to another [1]. Leprosy is more than a biological disease and is featured by stigma in the society leading to treating the affected ones with negative attitude [4]. Higher the associated stigma, lesser will be the chance to detect the new cases of leprosy early.

Despite being curable, each year globally around 200000 new cases of leprosy are detected [5]. Leprosy remains to be one of the neglected tropical diseases of developing countries in Africa and Asia with its burden being concentrated in Indonesia, Brazil and India. These three countries respectively contributed to 8%, 13% and 60% of the global new cases burden in 2015 while Nepal contributed to 1.3% [5; 6]. According to WHO factsheet, globally 210,758 new leprosy cases were detected in 2015 with prevalence rate of 0.29/10000 population [6]. However, the prevalence rate of leprosy in South-East Asian Region was 0.61/10000population [6]. Many countries have some areas of high endemicity showing high notification rates for new cases and witnessing continued transmission of leprosy [7]. Moreover, the open border between Nepal and India allows free migration of the population including leprosy affected persons. This may impede the early case detection and treatment.

Unfortunately, disability, disfigurement and the stigma associated with the leprosy have sustained and enhanced the stigma towards leprosy which in turn leads the affected ones to isolation, status concealment, delayed diagnosis and treatment. Leprosy affected ones in the early phase of the disease are generally suspicious of the diagnosis but fearing social isolation, leading to hesitancy towards seeking the advice and health care services [8]. A study done in Lalitpur Nepal in 1993 to 1995 showed that 6% (10/166) of leprosy affected persons reported of not seeking treatment earlier due to fear and social consequences including isolation [9]. Similarly, a quantitative study conducted in western Nepal in 2013 revealed that 66% of 135 leprosy affected persons intended to conceal their disease [10]. Moreover, in-depth interviews showed that 70% of 20 leprosy affected individuals intended to conceal their disease status with the major reasons being the fear of transmission, fear of exclusion, separation and rejection from the society [11]. Additionally, it has been observed that the social integration of people diagnosed of having leprosy is threatened when other people in the community come to know about it which results into applying the principle of silence and concealment of the disease status [12]. However, it is a serious public health problem since the multidrug therapy treatment should be initiated as soon as possible to prevent the disease progression resulting grade-2 disability which can pose further burden and severity condition in the lives of affected individuals [8; 13]. In order to control the cycle of concealment that leads to delayed health seeking and may lead to development of impairment and disability, early identification and treatment is critical.

Under leprosy control programme, Nepal declared that leprosy is no longer a public health problem in 2010 with the achievement of leprosy elimination in 2009 [14; 15]. However, there remain the challenges of sustaining this achievement and reducing the disease burden through quality services including early detection and prompt treatment [14]. On the contrary to decreasing incidence and prevalence of the disease, it has increased from 0.77 to 0.79, 0.84, 0.82, and 0.83 respectively during 2010 to 2014. Moreover, the country has been detecting more than 3000 new cases of leprosy annually [14]. Additionally, 18 districts of the nation have still prevalence above elimination level (prevalence rate of <1 case per 10000 population) and these districts contribute to 75% of the total incidence and accounts for around 3200 new cases of leprosy each year [15]. Most of these high prevalent districts are located in terai region of Nepal.

Despite the World Health Organization’s (WHO) target to eradicate leprosy by 2020, in the fiscal year 2016/2017, 19.77 leprosy affected individuals were diagnosed in every 10,000 population in the high prevalent districts of Terai regions of Nepal that are worst-hit by the burden of leprosy [16]. Towards increasing burden of leprosy in these regions, it has been argued that lack of awareness, poor personal hygiene, poor sanitation and low economic status of the people may be the reasons [17]. Additionally, people visit hospitals when the disease conditions get too worse; may it be due to inadequate awareness or lack of awareness towards skin-related diseases and its mode of transmission [17]. So, in order to address leprosy, better understanding about its cause, means of transmission and nature, and associated stigma is required.

In addition, to better understand leprosy and its social consequences it is important to study it in context, may it be the socio-cultural factors, belief systems, geography, economy, available resources or services [12]. The study done in Eastern Nepal revealed that leprosy affected individuals still encounter many constraints and restraints in their social life making them left out [4]. This fear of getting isolated may result in delayed in health care seeking [4]. Furthermore, a study in Nepal showed that majority of the respondent did not understand the cause of leprosy and were not aware of the duration of its treatment [18]. The study also emphasized the need of strengthening public/community awareness program towards leprosy [18]. According to a study done in Myanmar, it was found that community members were not sure about the cause of leprosy [19]. A study conducted in Pakistan revealed that more than one-fifth of the doctors did not have good knowledge regarding leprosy [20]. One of the major contributing factors towards the late diagnosis of leprosy is communities’ lack of knowledge regarding leprosy ultimately leading to increased likelihood of physical disability [2]. The available literatures have indicated that though leprosy is an old disease in terms of human civilization, it remains to be misunderstood and stigmatized. The knowledge and attitude of community towards leprosy remains poor which is mirrored by the study done in Cameroon which showed that less than one-fifth of the respondents knew the cause of leprosy and only about two-fifth of the respondents would shake hands with someone who is affected with leprosy [21].

A recent increase in new cases of leprosy from the Terai districts of Nepal has implications for the community where they live. How community members perceive leprosy affected persons and their attitude can affect their disease confession and health seeking at the hospital. Although studies in past have explored factors affecting community stigma towards leprosy in western and eastern Nepal, none in our knowledge has explored the community’s knowledge and attitude towards leprosy in Central southern Nepal. The main objective of this study was to assess the knowledge, attitude and stigma of leprosy amongst the community members living in Dhanusha and Parsa districts of Southern Central Nepal.

## Methods

### Study design

This was a cross-sectional study carried out amongst the community members living around teaching hospital (National Medical College and Teaching Hospital) of Parsa and Lalgadh Leprosy Hospital of Dhanusha district of state 2 of Nepal.

#### Study setting

State 2 of Nepal is a province situated in southeastern region of Nepal bordering State 1 and State 3 of Nepal to the North and Indian state of Bihar to the south. It includes eight districts from Saptari in the east to Parsa in the west. The other six districts include Bara, Rautahat, Sarlahi, Mahottari, Dhanusha and Siraha. It is the smallest state of Nepal by area and is second most populous state of Nepal. Slightly more than a half (50.3%) of the population of this state is male as per census 2011. Similarly,*Yadav*, *Teli*, *Koiri*, *Chamar*, *Musalman etc* are the major caste/ethnicity followed by people of state 2 of Nepal. Maithili, Bhojpuri and Nepali are the major languages spoken in this state. The literacy rate in the state is 49.54%. However, only 38.8% of female are literate. The major religion followed by people in state 2 of Nepal is Hinduism followed by Islam. The major occupation followed in state 2 of Nepal is agriculture as per census 2011 [22; 23].

The community-based study was conducted in July and August 2018 in two districts- namely Parsa and Dhanusha of state 2 of Nepal both of which sharing open border with Bihar state of India. These Terai districts have high prevalence of leprosy. The survey was carried out in communities surrounding National Medical College Teaching Hospital of Parsa (Bhediyahi) and in communities surrounding Lalgadh Leprosy Hospital (Mithila).

### Study respondents

Respondents of both sexes aged between 18 years and 60 years were involved in the survey. However, individuals who were having hearing impairment and mental illness were excluded from the survey.

### Sample size determination

The sample size was calculated by using StatCalc Epi-Info. With prevalence 50% and margin of error 5%, the sample size was 384. Assuming the non-response of 10%, the final sample size calculated was 423.

### Sampling technique

The data was collected in two priority districts of state 2 of Nepal viz Parsa and Dhanusha. From both districts the communities (Bhediyahi and Mithila) surrounding the teaching hospital and leprosy hospital of Parsa and Dhanusha respectively were chosen for data collection. From Bhediyahi 212 and from Mithila 211 households were taken systematically. From each selected household one eligible respondent of age in between 18 and 60 years who gave his/her consent to participate in the study were selected for interview.

### Data collection tool

Structured questionnaire consisting of four parts was prepared after review of relevant literatures. The first part of the questionnaire was related to socio-demographic characteristics of the study participants, the second part was related to assessment of community member’s knowledge regarding leprosy, the third part was related to assessment of community member’s attitude towards leprosy and leprosy patients, and the fourth part was related to assessment of stigma attached in community towards leprosy and leprosy patients. The fourth part, EMIC scale (The Explanatory Model Interview Catalogue) is a reliable and validated tool to assess the community stigma towards leprosy (Rensen et al) [24]. The Nepali version of EMIC scale has been used in the past by one study conducted in western Nepal [25]. The other parts of the structured questionnaire in English were translated into Nepali language so that it can be relevantly used in the Nepalese context. A back-translation was then done to English language. The back translation of the tool from Nepali to English language was blind to the original questionnaire. Then the translated questionnaire in Nepali and back-translated questionnaire in English and the original questionnaire were all reviewed by assessing the meaning of each word to ensure the accuracy of the translation and the final questionnaire in Nepali was prepared.

### Data collection procedure

Data was collected by the researchers themselves through face to face interview. The data was collected from the members of each selected household whose age was greater than 18 years old and less than 60 years and who gave his/her voluntary consent. The purpose of data collection was explained first to respondents to increase their awareness about the study before the start of the interview. They were informed regarding their voluntary participation in the study and their right to not answer any questions they did not want to. They were also ensured about regarding maintaining confidentiality of the information they provided as the researchers neither asked their name nor recorded any kind of respondent personal identity which could identify their name.

### Operational definitions

Knowledge of leprosy- Based on reported response each correct response towards each item of the knowledge questionnaire; the level of knowledge towards leprosy was assessed. Altogether sixteen self-reported items were considered for assessing level of knowledge which included questions with one correct answer as well as questions of dichotomous response (Yes/No) like hearing about leprosy, knowing its cause, sign and symptoms, leprosy as very infectious disease, its transmission, is it treatable, and its treatment. The level of knowledge was categorized as having good knowledge or poor knowledge. Good knowledge of leprosy- Respondents who were able to answer 75% or more of knowledge questions correctly were regarded as having good knowledge of leprosy. Poor knowledge of leprosy- Respondents who were able to answer less than 75% of knowledge questions correctly were regarded as having poor knowledge of leprosy. Additionally, the source of information on leprosy, knowledge regarding leprosy being a severe disease, and knowledge regarding first sign and symptoms of leprosy were also assessed. Attitude towards leprosy- It referred to community member’s perception towards leprosy and/or leprosy affected individuals. Attitude was assessed through 13 statements (10 positive statements towards leprosy and 3 negative statements towards leprosy) with response either ‘Yes’ or ‘No’. A response with ‘Yes’ towards each positive statement was given a score of 1 and a response with ‘No’ towards each positive statement was given a score of 0. Similarly, a response with ‘No’ towards each negative statement was given a score of 1 and a response with ‘Yes’ towards each negative statement was given a score of 0. Attitude was categorized as either having favorable attitude towards leprosy or unfavorable attitude towards leprosy based on individual respondent’s attitude score. Favorable attitude towards leprosy- Respondents who scored attitude score 7 or more (>50% of maximum attitude score) were regarded as having favorable attitude towards leprosy. Unfavorable attitude towards leprosy- Respondents who scored attitude score less than 7(<50% of maximum attitude score) were regarded as having unfavorable attitude towards leprosy. Level of stigma towards leprosy- The level of stigma towards leprosy was assessed based on respondent’s individual EMIC score. The EMIC score was calculated based on individual responses towards 15 items of the EMIC scale which is a standard tool to assess stigma towards leprosy. Each item/question in the scale was scored on the basis of response as “Yes = 2, Possibly = 1, No or Don’t know = 0”. Further, the level of stigma was assessed based on calculated individual respondent’s EMIC score and was categorized as high level of stigma, moderate level of stigma and low level of stigma. The category of the level of stigma towards leprosy was adapted. Respondents who scored EMIC score greater than 20 were regarded as having high level of stigma towards leprosy, respondents who scored EMIC score in the range of 10–20 were regarded as having moderate level of stigma towards leprosy, and respondents who scored EMIC score in the range of 0–10 were regarded as having low level of stigma towards leprosy.

### Data processing and analysis

The collected data were checked daily for completeness and consistency before data processing and analysis. The collected data was cleared, checked and analyzed by using tally sheet and computer. Data was entered and analyzed in SPSS version 20. Both descriptive and statistical inferences were used to analyze the data. Descriptive statistics like frequency, percentage, and median were used to describe the socio-demographic characteristics, level of knowledge, level of attitude and level of stigma of the study participants. Proportions were calculated, and the Chi-square test was used to examine relationship between socio-demographic characteristics and level of knowledge; socio-demographic characteristics and level of attitude; and level of knowledge and level of attitude. Mann Whitney U test and Kruskal Wallis H test were used to analyze the difference in total perceived stigma score using EMIC between different socio-demographic characteristics of the community. Further, binary logistic regression analysis was carried out to determine predictors of unfavorable attitude towards leprosy.

### Ethics statement

Ethical clearance was obtained from Institutional Review Committee (IRC) of National Medical College (FNMC-310-074-075). Further, for each study participants, the purpose of the study was stated by the researchers prior to data collection. In addition, participants were informed that they have full right to refuse participating in the study and can interrupt the interview if not comfortable with it. However, they were informed that their participation in the study is very important. Participation of each respondent in this study was voluntary and data was collected from each participant once they gave an informed consent. Confidentiality of the information was maintained, and anonymity of the study participants was respected during data processing and analysis.

## Results

### Socio-demographic characteristics of the study participants

Four hundred and twenty-three (423) individuals were contacted and interviewed in the survey with age ranging between 18 years and 60 years, with around 36% above 40 years and 29% below 24 years. They were 58.6% males, 34.8% *Brahmin/Chhetri* (considered to be higher class as per ethnicity in Nepal) and 69.7% married. Almost half of the respondents (49.9%) were from nuclear family. More than one-third of the participants (40.9%) had bachelors level or higher degree of education. Most of them were service holder (45.6%) followed by farmer (24.3) and with monthly income more than Nepalese Rupees twenty thousand (49.2%).

### Participants’ knowledge of leprosy

All the study participants had heard about leprosy. More than 4/5^th^ of the participants (88.4%) reported of knowing the cause of leprosy. More than 3/4^th^ of the study participants (79.4%) believed leprosy to be highly infectious disease. Similarly, 69% of them reported of knowing how leprosy is transmitted. Also, 81.1% of the study participants reported of knowing the signs and symptoms of leprosy. With regards to the first sign and symptom of the disease, 46.8% of the participants reported skin involvement, 3.1% reported nerve involvement and 31.2% reported both skin and nerve involvement as the first sign of the disease.

Although 88.4% responded of knowing the cause of leprosy, only 62.6% of them reported of bacteria being the cause of leprosy. Surprisingly, 21.1% of them reported bad blood as the cause of leprosy, followed by curse by god (8.8%), heredity (3.2%), bad deeds (2.7%), and unclean environment (1.6%). However, only 43.8% responded that leprosy is transmitted by prolonged close contact with leprosy affected individuals. Majority of the participants (87.7%) thought leprosy to be a curable disease. But, 25.7% of them reported religious rituals as the treatment for leprosy. In addition, most of the participants (65.2%) thought leprosy to be a severe disease. Nevertheless, only 62.2%, 48.9%, 30%, and 66.7% of them reported skin patches, loss of sensation, deformity and ulcer respectively to be the signs and symptoms of the disease. Surprisingly, 28.4% and 31.2% of the participants also responded tingling and skin irritation respectively to be the signs and symptoms of leprosy. Around 2/5^th^ of the study respondents (38.8%) said that they would not go to hospital or doctor if they get to know of having leprosy. Based on correct response towards the questions related to knowledge, it was found that 57.9% of the study participants had poor knowledge of leprosy and remaining (42.1%) of them had good knowledge of leprosy.

### Source of information of leprosy

The major source of information about leprosy for the community people was found to be hospital and health worker comprising (33.1%) followed by media (30.7%).

### Relationship between knowledge of leprosy and socio-demographic characteristics

The knowledge of leprosy among community people were influenced by socio-demographic characteristics (Table 1). It was found that there was highly significant association between the level of knowledge of leprosy among study participants with age, sex, ethnicity, religion, educational status, occupation and monthly income (P< 0.001). Most of the study participants from older adult age group (>45 years) had good knowledge of leprosy while majority of female had poor knowledge of leprosy as compared to male. Most of the respondents from *Dalit* or *Janajati* background had poor knowledge of leprosy. Majority of the non-Hindu respondents had good knowledge of leprosy. Respondents with higher education including bachelor or master or higher degree were having good knowledge of leprosy. Respondents with occupation service had good knowledge of leprosy. Most of the individuals with monthly income more than Nepali Rupees 20000 (Nepali Rupees 20000 approximately equivalent to USD 178 as of 29 November 2018) had good knowledge of leprosy. The level of knowledge of leprosy was also significantly associated with type of family of the study participants where individuals from joint family had good knowledge of leprosy (P = 0.034). However, marital status was not found to influence the level of knowledge of leprosy (P = 0.101).

**Table 1:** Relationship between knowledge of leprosy and socio-demographic variables.

Characteristics/Variable	Category	N* of respondent	Poor Knowledge	Good Knowledge	P-value*
Age (in years)	< = 24	123(29%)	95(22.5%)	28(6.5%)	**P < 0.001**
	25–29	46(10.87%)	21(5.96%)	25(4.91%)	
	30–34	34(8.03%)	21(5.96%)	13(2.07%)	
	35–39	65(15.36%)	50(11.82%)	15(3.54%)	
	40–44	71(16.78%)	28(6.62%)	43(10.16%)	
	> = 45	84(19.86%)	30(7.09%)	54(12.77%)	
Sex	Female	175(41.4%)	128(30.3%)	47(11.1%)	**P < 0.001**
	Male	248(58.6%)	117(27.7%)	131(30.9%)	
Ethnicity	*Dalit/Janajati*	124(29.3%)	102(24.1%)	22(5.2%)	**P < 0.001**
	Other Backward Caste and Minorities	152(35.9%)	80(18.9%)	72(17%)	
	*Brahmin/Chhetri*	147(34.8%)	63(14.9%)	84(19.9%)	
Religion	Hindu	353(83.5%)	238(56.3%)	115(27.2%)	**P < 0.001**
	Muslim/Christian/Other	70(16.5%)	7(1.6%)	63(14.9%)	
Marital Status	Married	295(69.7%)	161(38.1%)	134(31.6%)	P = 0.101
	Unmarried	84(19.9%)	56(13.2%)	28(6.7%)	
	Widow/Widower/Separated/Divorced	44(10.4%)	28(6.6%)	16(3.8%)	
Type of Family	Nuclear	211(49.9%)	133(31.4%)	78(18.5%)	**P = 0.034**
	Joint	212(50.1%)	112(26.5%)	100(23.6%)	
Educational Status	Not attended school	9(2.1%)	9(2.1%)	0	**P < 0.001**
	Informal education	20(4.7%)	20(4.7%)	0	
	Primary level	69(16.3%)	69(16.3%)	0	
	Lower secondary level	51(12.1%)	51(12.1%)	0	
	Secondary and higher secondary level	101(23.9%)	57(13.5%)	44(10.4%)	
	Bachelors and/or above	173(40.9%)	39(9.2%)	134(31.7%)	
Occupation	Farmer	103(24.4%)	73 (17.3%)	30(7.1%)	**P < 0.001**
	Laborer	24(5.7%)	24(5.7%)	0	
	Business	25(5.9%)	22(5.2%)	3(0.7%)	
	Service	193(45.6%)	76(17.9%)	117(27.7%)	
	Housewife	48(11.3%)	43(10.2%)	5(1.1%)	
	Others	30(7.1%)	7 (1.7%)	23 (5.4%)	
Monthly Income in NRs	8000–12000	61(14.4%)	61(14.4%)	0	**P < 0.001**
	12000–16000	15(3.5%)	15(3.5%)	0	
	16000–20000	139(32.9%)	87(20.6%)	52(12.3%)	
	> = 20000	208(49.2%)	82(19.4%)	126(29.8%)	

*p-value calculated using Chi-square test.

### Participants’ attitude towards leprosy

Around 3/5^th^ of the study participants (59.1%) had unfavorable attitude towards leprosy and 40.9% had favorable attitude. Most of the participants (51.8%) responded they would sit together with leprosy affected individuals in public conveyance, 51.3% said that they would not avoid having food or other activities with leprosy patients, would agree to work in the same environment with leprosy affected ones (52.5%), would not feel shame to share the status to others if anyone in the family had leprosy (53.4%). More than 4/5^th^ (96.2%) reported that they would support leprosy affected ones if they would need it. However, only 12.5% reported that they would share foods with leprosy patients, only 32.6% would take cooked foods by the leprosy affected individuals, and majority of them reported that they would not marry individuals from family with history of leprosy (82%). Similarly, majority of them (84%) reported that it is difficult for anyone with leprosy to get married.

### Relationship between socio-demographic characteristics and attitude towards leprosy

The level of attitude towards leprosy among community members were found to be influenced by socio-demographic variables (Table 2). It was found that there was highly significant association between level of attitude towards leprosy and age, sex, ethnicity, religion, marital status, educational status and occupation (P<0.001). Most of the study participants from older adult age group (>45 years) had favorable attitude towards leprosy while majority of female had unfavorable attitude towards leprosy as compared to male. Most of the respondents from *Dalit* or *Janajati* background had unfavorable attitude towards leprosy. All of the non-Hindu respondents had favorable attitude towards leprosy. Majority of the married respondents had unfavorable attitude towards leprosy. Respondents with higher education including bachelor or master or higher degree were having favorable attitude towards leprosy. Majority of the housewives were found to have had unfavorable attitude towards leprosy.

**Table 2:** Relationship between socio-demographic variables and attitudes.

Characteristics/Variable	Category	N* of respondents	Unfavorable Attitude	Favorable Attitude	P-value*
Age (in years)	< = 24	123(29%)	98(23.2%)	25(5.8%)	**P < 0.001**
	25–29	46(10.87%)	39(9.21%)	7(1.66%)	
	30–34	34(8.03%)	21(4.96%)	13(3.07%)	
	35–39	65(15.36%)	40(9.45%)	25(5.91%)	
	40–44	71(16.78%)	40(9.45%)	31(7.33%)	
	> = 45	123(29%)	12(2.8%)	72(26.2%)	
Sex	Female	175(41.4%)	130(30.7%)	45(10.4%)	**P < 0.001**
	Male	248(58.6%)	120(28.4%)	128(30.2%)	
Ethnicity	*Dalit/Janajati*	124(29.3%)	107(25.3%)	17(4%)	**P < 0.001**
	Other Backward Caste and Minorities	152(35.9%)	88(20.8%)	64(15.1%)	
	*Brahmin/Chhetri*	147(34.8%)	55(13%)	92(21.8%)	
Religion	Hindu	353(83.5%)	250(59.1%)	103(24.4%)	**P < 0.001**
	Muslim/Christian/Others	70(16.5%)	0	70(16.5%)	
Marital Status	Married	295(69.7%)	191(45.2%)	104(24.5%)	**P < 0.001**
	Unmarried	84(19.9%)	59(13.9%)	25(6%)	
	Widow/Widower/Separated/Divorced	44(10.4%)	0	44(10.4%)	
Educational Status	Not attended school	9(2.1%)	9(2.1%)	0	**P < 0.001**
	Informal education	20(4.7%)	20(4.7%)	0	
	Primary level	69(16.3%)	41(9.7%)	28(6.6%)	
	Lower secondary level	51(12.1%)	51(12.1%)	0	
	Secondary and higher secondary level	101(23.9%)	60(14.2%)	41(9.7%)	
	Bachelors and/or above	173(40.9%)	69(16.3%)	104(24.6%)	
Occupation	Farmer	103(24.4%)	45(10.6%)	58(13.8%)	**P < 0.001**
	Labourer	24(5.7%)	24(5.7%)	0	
	Business	25(5.9%)	22(5.2%)	3(0.7%)	
	Service	193(45.6%)	106(25.1%)	87(20.5%)	
	Housewife	48(11.3%)	43(10.2%)	5(1.1%)	
	Others	30(7.1%)	10(2.4%)	20(4.7%)	

*p-value calculated using Chi-square test.

### Independent predictors of attitude towards leprosy

The binary logistic regression showed that the individuals who knew how leprosy is transmitted are likely to have 3.35 times favorable attitude of sitting together with leprosy affected individuals in the public conveyance (Table 3). Also, those who think leprosy to be very infectious would have 2.1 times higher chance of staying far away from leprosy patients. In addition, those who know it is transmitted by prolonged close contact only would have 13 times higher chance of letting own child to play with children of leprosy affected individuals.

**Table 3:** Independent predictors of attitude towards leprosy.

Attitudes	Independent Predictors		95% CI		
		OR	Lower	Upper	P-value*
Would sit together with leprosy patient in public conveyance	Know how leprosy is transmitted	3.35	2.01	5.57	**P <0.001**
Would stay far away from leprosy patient	Think leprosy is very infectious	2.11	1.07	4.16	**P = 0.03**
Would allow own child to play with children of leprosy patients	Thinks leprosy is transmitted by prolonged close contact	13.7	8.26	22.7	**P <0.001**

*p-value calculated using binary logistic regression.

### Relationship between level of knowledge and level of attitude towards leprosy

The Chi-square test showed that the attitude was highly influenced by the knowledge of leprosy among the community members (Table 4). There was highly significant association between level of attitude and level of knowledge of leprosy among the study participants (P<0.001). The finding revealed that better the knowledge of leprosy among individuals, more the chance of having positive attitude towards leprosy and leprosy patients.

**Table 4:** Relationship between level of knowledge and level of attitude.

Characteristics/Variable	Category	N* of respondents	Unfavorable Attitude	Favorable Attitude	P-value*
Level of knowledge	Poor knowledge	245 (57.9%)	210 (49.7%)	35 (8.2%)	**P < 0.001**
	Good knowledge	178 (42.1%)	40 (9.5%)	138 (32.6%)	

*p-value calculated using Chi-square test.

### Stigma towards leprosy

The EMIC profile of the study participants revealed that 44% were having high stigma, 33.3% were having moderate level of stigma and 22.7% were having low stigma towards leprosy and leprosy patients.

### Perceived stigma towards leprosy

**Fig 1:**
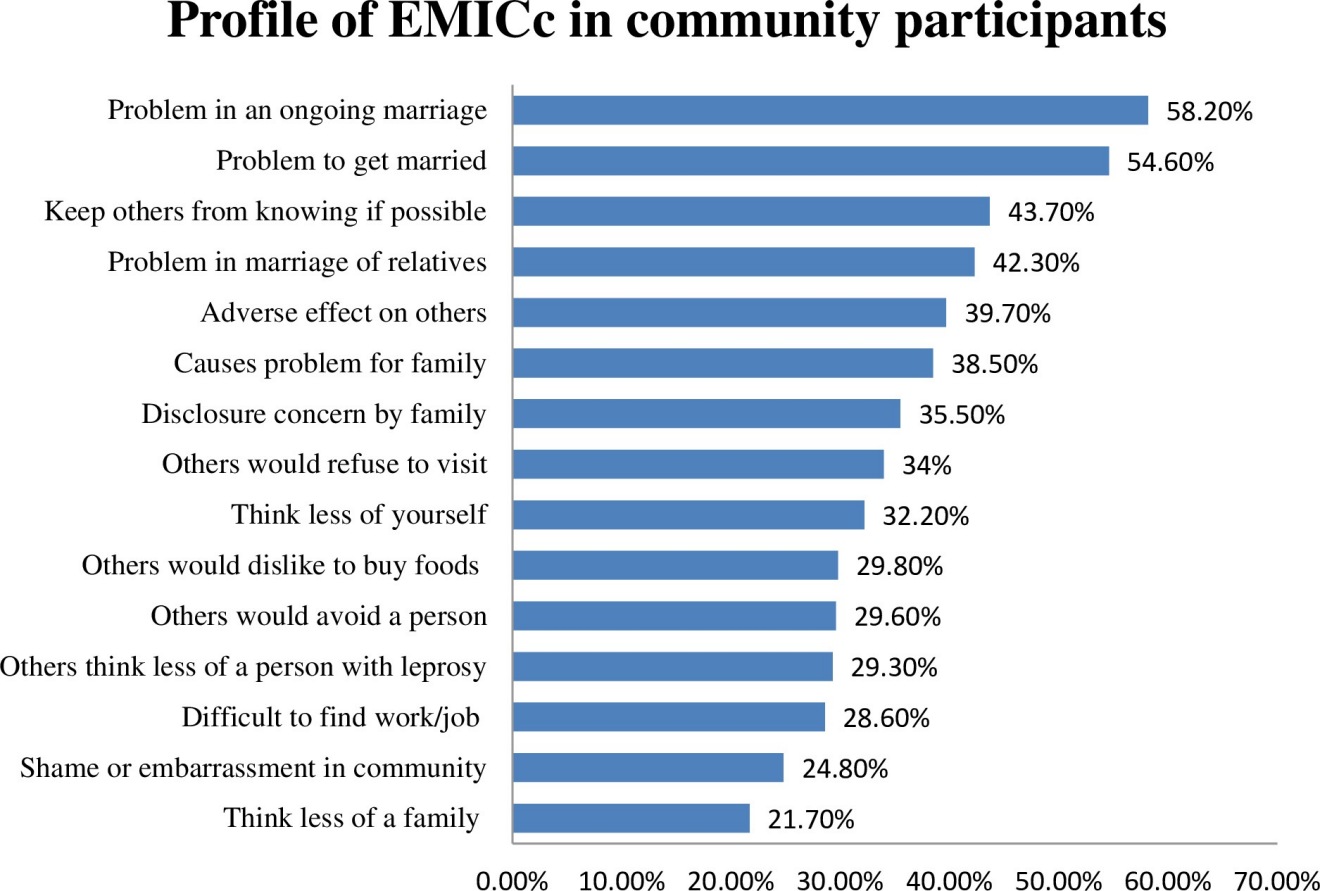
Statement wise stigma statement with percentage answering “Yes”.

The assessment of EMIC score was done to measure the perceived stigma towards leprosy and leprosy patients in community members. The median score was calculated to analyze the difference of stigma between various groups. It was found that 43.7% of the study participants would keep others from knowing leprosy status if possible, 32.2% would think less of self due to leprosy affected individual in the family, 24.8% think that leprosy has caused shame or embarrassment in the community, 29.3% feel others think less of a person with leprosy, 39.7% think that there would be adverse effect on others if they know someone’s status of leprosy, and 29.6% think that others would avoid a person with leprosy (Fig 1).

### Relationship between socio-demographic and Knowledge variables and EMIC score

It was found that there was highly significant association between EMIC score and age, ethnicity, marital status, educational status, occupation, monthly income, knowledge of leprosy transmission, knowledge of cause of leprosy, sex, religion, and income sufficiency for living, knowledge regarding leprosy is treatable, Knowledge of sign and symptoms of leprosy (P<0.001) (Table 5, Table 6, Table 7). The finding showed that perceived stigma towards leprosy (EMIC score) was lower at increasing age > 40 years. EMIC score was high among *Dalit/Janajati*, unmarried, female, Hindu participants, and participants with insufficient income for living. Similarly, study participants who did not have knowledge of leprosy transmission and who did not know the cause of leprosy had high EMIC score. Further, the study participants who thought leprosy as not curable disease were found to have had high EMIC score. Furthermore, respondents who knew loss of sensation, deformity and ulcer as sign of leprosy had low EMIC score while respondents who knew skin patch as sign of leprosy had high EMIC score. Respondents who though skin itchiness as sign and symptom of leprosy had high EMIC score. Additionally, the study participants who correctly knew bacteria as the cause of leprosy and prolonged close contact being the means to transmit leprosy had low EMIC score. Similarly, respondents with higher educational status (Bachelor’s degree and above), engaged in service and with monthly income of >20000 NRs were found to have had low EMIC score. However, there was no association between EMIC score and type of family (P = 0.177), residence or district (P = 0.56), knowledge regarding leprosy being an infectious disease (P = 0.551), knowledge regarding leprosy being a severe disease (P = 0.51) and knowing tingling as a sign of leprosy (P = 0.133). These factors (type of family, residence, knowledge regarding leprosy being a severe disease and knowing tingling as a sign of leprosy) were found to have no influence on perceived stigma towards leprosy in community people.

**Table 5:** Relationship between socio-demographic variables and EMIC score.

**Characteristics**	N*of Respondents	**Median**	P-value*
**Age in years**			
**≤ 24**	123 (29%)	22	**P < 0.001**
**25–29**	46 (10.87%)	16	
**30–34**	34 (8.03%)	23	
**35–39**	65 (15.36%)	17	
**≥ 40**	155 (36.64%)	10	
**Ethnicity**			
***Dalit/Janajati***	124 (29.3%)	22	**P < 0.001**
**Other Backward Caste and Minorities**	152 (35.9%)	13	
***Brahmin/Chhetri***	147 (34.8%)	16	
**Marital Status**			
**Married**	295 (69.7%)	16	**P < 0.001**
**Unmarried**	84 (19.9%)	25	
**Widow/Widower/Separated/Divorced**	44 (10.4%)	21	
**Educational status**			
**Not attended school**	9 (2.1%)	22	**P < 0.001**
**Informal education**	20 (4.7%)	24	
**Primary level**	69 (16.3%)	22	
**Lower secondary level**	51 (12.1%)	26	
**Secondary and higher secondary level**	101 (23.9%)	17	
**Bachelors and/or above**	173 (40.9%)	10	
**Occupation**			
**Farmer**	103 (24.4%)	21	**P < 0.001**
**Laborer**	24 (5.7%)	27	
**Business**	25 (5.9%)	17	
**Service**	193 (45.6%)	12	
**Housewife**	48 (11.3%)	22	
**Others**	30 (7.1%)	18	
**Monthly Income in NRs**			
**8000–12000**	61 (14.4%)	26	**P < 0.001**
**12000–16000**	15 (3.5%)	22	
**16000–20000**	139 (32.9%)	21	
**> = 20000**	208 (49.2%)	12	

*p-value calculated using Kruskal Wallis H test.

**Table 6:** Relationship between socio-demographic and Knowledge variables and EMIC score.

**Characteristics**	N*of Respondents	**Median**	P-value*
**Sex**			
**Female**	175 (41.4%)	22	**P < 0.001**
**Male**	248 (58.6%)	12	
**Religion**			
**Hindu**	353 (83.5%)	21	**P < 0.001**
**Muslim/Christian/Others**	70 (16.5%)	8	
**Type of family**			
**Nuclear**	211 (49.9%)	16	P = 0.177
**Joint**	212 (50.1%)	21	
**District**			
**Dhanusha (with leprosy hospital)**	212 (50.1%)	17	P = 0.56
**Parsa (with medical teaching hospital)**	211 (49.9%)	17	
**Income sufficient for living**			
**Yes**	257 (60.8%)	12	**P < 0.001**
**No**	166 (39.2%)	24	
**Know cause of leprosy**			
**Yes**	374 (88.4%)	16	**P < 0.001**
**No**	49 (11.6%)	24	
**Know transmission of leprosy**			
**Yes**	292 (69%)	14	**P < 0.001**
**No**	131 (31%)	22	
**Leprosy is very infectious**			
**Yes**	292 (69%)	22	P = 0.551
**No**	131 (31%)	14	
**Leprosy is treatable**			
**Yes**	371 (87.7%)	16	**P < 0.001**
**No**	52 (12.3%)	25	
**Leprosy is severe disease**			
**Yes**	340 (80.4%)	16	P = 0.51
**No**	83 (19.6%)	24	
**Knows sign and symptoms of leprosy**			
**Yes**	343 (81.1%)	16	**P < 0.001**
**No**	80 (18.9%)	12	
**Skin patches as a sign of leprosy (N = 343)**			
**Yes**	263 (62.2%)	15	**P < 0.001**
**No**	80 (18.9%)	16	
**Tingling as a sign of leprosy (N = 343)**			
**Yes**	120 (28.4%)	16	P = 0.133
**No**	223 (52.7%)	12	
**Skin itchiness as a sign of leprosy (N = 343)**			
**Yes**	132 (31.2%)	21	**P < 0.001**
**No**	211 (29.9%)	10	
**Loss of sensation as sign of leprosy (N = 343)**			
**Yes**	207 (48.9%)	10	**P < 0.001**
**No**	136 (32.2%)	21	
**Deformity as a sign of leprosy (N = 343)**			
**Yes**	127 (30%)	4	**P < 0.001**
**No**	216 (51.1)	18	
**Ulcer as a sign of leprosy (N = 343)**			
**Yes**	282 (66.7%)	12.5	**P < 0.001**
**No**	61 (14.4%)	16	
**Go to hospital/doctor if affected with leprosy**			
**Yes**	259 (61.2%)	12	**P < 0.001**
**No**	164 (38.8%)	22	

*p-value calculated using Mann Whitney U test.

**Table 7:** Relationship between knowledge and EMIC score.

Characteristics	N*of Respondents	Median	P-value*
How is leprosy transmitted (N = 292)			**P < 0.001**
From animal	10 (3.4%)	16	
From mosquito	28 (9.6%)	21	
Air or flatus of leprosy patients	23 (7.9%)	23	
Sexual contact with leprosy patients	42 (14.4%)	22	
Prolonged close contact with leprosy patients	128 (43.8%)	8.5	
Sitting close with leprosy patients	50 (17.1%)	26	
Sharing personal items with leprosy patients	11 (3.8%)	5	
What is the cause of leprosy? (N = 374)			**P < 0.001**
Bacteria or any microorganism	234 (62.6%)	10	
Curse by god	33 (8.8%)	26	
Bad deeds/*Karma*	10 (2.7%)	23	
Unclean environment	6 (1.6%)	25	
Hereditary	12 (3.2%)	15	
Bad blood	79 (21.1%)	22	
First sign of leprosy (N = 343)			**P < 0.001**
Skin involvement	198 (57.7%)	21	
Nerve involvement	13 (3.8%)	9	
Both skin and nerve involvement	132 (38.5%)	5	

*p-value calculated using Kruskal Wallis H test.

## Discussion

The overall findings of the study revealed that only 42.1% of the community people had good knowledge of leprosy with major source of information being local health worker and media (63.8%). Similarly, only 40.9% of the study respondents were found to have favorable attitude towards leprosy. Additionally, it was also found that the community-based stigma towards leprosy and leprosy affected persons is still prevalent among study participants living in the study districts- Dhanusha and Parsa.

The findings of this community-based study showed that still around 3/5^th^ of the study participants had poor knowledge of leprosy. This result is supported by the study done in eastern Nepal [4]. Similarly, the study done in western Nepal also showed similar result with almost half having some kind of knowledge on leprosy cause, transmission and clinical manifestation [25]. This finding is also congruent to the study done in community members of Andhra Pradesh and Orissa which showed that 35–50% of the respondents had high level knowledge of leprosy [26]. However, a study done in Indian rural community to assess knowledge and attitude towards leprosy after post elimination phase showed that 78.94% of the respondents had good knowledge and 69% had positive attitude towards leprosy [27]. Similarly, a study done in Ethiopia also revealed worse scenario with around 80% of the respondents having low level of knowledge of leprosy [28]. The reason for this difference in knowledge and attitude may be due to the different socio-cultural context in relation to Terai districts of Nepal.

This study and Ethiopian study resembles in relation to the response that 100% of the participants had heard about leprosy [28]. However, the study done in Cameroon showed that only 82.4% of the respondents had heard about leprosy [21]. Apart from responding bacteria as the cause of leprosy, participants also responded wrongly citing god’s sin, bad deeds, bad blood, and heredity as the causes of leprosy which is similar to the findings of the study done in Cameroon and Ethiopia [21; 28]. This finding has also been supported by the study done in Uttar Pradesh, India [29]. These myths are rooted in the socio-cultural context of the communities in Asia, Africa and South America as evident in the literature written by Wong and Subramaniam [30].

The current study revealed that around 3/5^th^ of the respondents had unfavorable attitude towards leprosy. The findings related to prevalence of unfavorable attitude such as eating limitation and negative behavior in the community are consistent to the study done in eastern Nepal [4]. One of the reasons behind this unfavorable attitude may be overall poor literacy rate and more specifically poor literacy rate of female of the study region. The level of attitude among the community members towards leprosy is also similar in the study done in Ethiopia [28].

The study findings showed that knowledge and attitude of leprosy among community are influenced by various socio-demographic characteristics of the community members. This finding is supported by the study done in community members in western region of Nepal [25]. This result is also congruent with the studies done in Cameroon and Ethiopia [21; 28].

This study showed that stigma is still prevalent in communities of Terai districts of Nepal. The study identified various kinds of stigma/myths such as participants preferring to hide their leprosy status, thinking less of self if any of the family member is affected by leprosy, thinking that leprosy has caused shame, feeling others think less of a person with leprosy, thinking that others would avoid a person with leprosy, and reporting that it would cause problem in marriage. This indicated that preference to concealment towards leprosy status is still prevalent due to grounded stigma of leprosy in communities of Nepal. This finding is similar to study done in Western region of Nepal [25]. Such finding relating to prevalence of preference towards concealment of the disease and feeling of shame towards leprosy is also congruent to studies done in eastern Nepal [4; 31]. The finding regarding stigma related to leprosy causing problem in marriage was similar to the qualitative study done in South East Nepal [32]. The results regarding the nature of perceived stigma towards leprosy and leprosy patients among community people was similar in the study conducted in Thailand [33]. The finding is also supported by the study done in Indonesia [34]. The level of stigma is high among high proportion of study participants in this study and this finding is supported by the studies done in western Nepal [25] and rural India [34], example around 47% and 22% of the respondents with response of not preferring to buying foods from leprosy affected individuals in western Nepal and rural India respectively which is quite similar to this study (29%). In this study, socio-demographic variables like age, ethnicity, marital status, education, occupation, etc. and knowledge were found to influence stigma and EMIC score among community members. The finding is supported by the findings of the study done in Pokhara [25]. Nevertheless, unlike the finding of the study done in Pokhara, there was no association between perceived stigma of leprosy and residence (districts) of the study participants [25]. The reason for this dissimilarity may be the effect of teaching hospital and leprosy hospital raising similar kind of consciousness towards leprosy among the people. Knowledge and beliefs about leprosy has been found to be associated with stigma in leprosy in many studies conducted in China [35] and Nepal [4]. Myths such as not allowing child to play with children of leprosy affected individuals, not sitting together with leprosy affected ones, not preferring to marry with one with family history of leprosy, and not sharing foods with leprosy affected individuals suggest how deep-rooted misconceptions of leprosy are prevalent in the communities. It was also found that study participants reported that leprosy affected ones would get difficulty in getting job.

There were various unfavorable attitudes towards leprosy prevalent in the communities of Terai districts of Nepal. Similarly, knowledge regarding leprosy causes, transmission, sign and symptoms and treatment were also not adequate for breaking the transmission of the disease and early identification for prompt treatment. Also, different myths and misconceptions are still present in the communities in different socio-demographic groups of the population. Progress towards leprosy eradication is only possible by making people to better understand its transmission. So, to make the leprosy control programme a success by eliminating and eradicating this old disease, the first and foremost thing to do is to strategize the programmes as per strata according to socio-demographic characteristics of the population for enhancing their knowledge regarding leprosy, its cause, symptoms, transmission, prevention and treatment and thereby changing the attitude to make it more favorable towards leprosy.

Furthermore, advocacy programmes should be developed engaging people affected with leprosy, local health workers, and deep-rooted traditional healers of the rural communities and local media to provide information about leprosy to the community people. Also, empowerment workshops should be organized for the leprosy affected individuals including unaffected females of the community who can further help to aware other people once empowered. Additionally, more information, education and communication materials need to be developed and made accessible to the general public in both the least and the most affected communities. Further studies are needed to develop new diagnostic and screening tools which can identify leprosy at earlier and hidden stage at the community level. As state 2 of Nepal lies adjacent to communities of India which is one of the countries with concentrated burden of leprosy, further studies on communities of state 2 of Nepal bordering to Bihar state of India is recommended which might show the issue of inadequate knowledge, negative attitude and high stigma at more worse scenario. These communities need to be addressed in terms of strengthening their capacity to prevent and control this growing burden of leprosy with sufficient supporting evidence. Further study on the issues of this neglected tropical disease in a larger scale both in rural and urban areas of Nepal is recommended to bring forth clearer picture.

## Supporting information

S1 Checklist STROBE checklist for this cross-sectional study. (DOCX)

S1 Text Questionnaire used for data collection in this study. (DOCX)
