# Supplementary material for: Community knowledge, attitude, and perceived stigma of leprosy amongst community members living in Dhanusha and Parsa districts of Southern Central Nepal
Source: PLoS Negl Trop Dis. 2019 Jan 11;13(1):e0007075. doi: 10.1371/journal.pntd.0007075 (PMC6329495; doi:10.1371/journal.pntd.0007075)
Supplement: S1 Checklist — (DOCX) [file pntd.0007075.s001.docx]

STROBE Statement—Checklist of items that should be included in reports of ***cross-sectional studies***

|  | Item No | Line number |
| --- | --- | --- |
| **Title and abstract** | 1 | Line 1-39 |
| Introduction | |  |
| Background/rationale | 2 | Line 59-144 |
| Objectives | 3 | Line 144-146 |
| Methods | |  |
| Study design | 4 | Line 148-151 |
| Setting | 5 | Line 152-168 |
| Participants | 6 | Line 169-172 |
| Variables | 7 | Line 184-200 |
| Data sources/ measurement | 8* | Line 201-248 |
| Bias | 9 |  |
| Study size | 10 | Line 173-183 |
| Quantitative variables | 11 | See item No 7 |
| Statistical methods | 12 | Line 249-262 |
| Results | |  |
| Participants | 13* | Line 274-282 |
| Descriptive data | 14* | Line 274-282 |
| Outcome data | 15* | Line 305-307, 331-332, 376-378 |
| Main results | 16 | Line 285-414 |
| Other analyses | 17 | Line 285-414 |
| Discussion | |  |
| Key results | 18 | Line 416-421 |
| Limitations | 19 | Line 510-514 |
| Interpretation | 20 | Line 422 -488 |
| Generalisability | 21 | Line 490-518 |
| Other information | |  |
| Funding | 22 |  |

*Give information separately for exposed and unexposed groups.

**Note:** An Explanation and Elaboration article discusses each checklist item and gives methodological background and published examples of transparent reporting. The STROBE checklist is best used in conjunction with this article (freely available on the Web sites of PLoS Medicine at http://www.plosmedicine.org/, Annals of Internal Medicine at http://www.annals.org/, and Epidemiology at http://www.epidem.com/). Information on the STROBE Initiative is available at www.strobe-statement.org.
