# Supplementary material for: Community knowledge, attitude, and perceived stigma of leprosy amongst community members living in Dhanusha and Parsa districts of Southern Central Nepal
Source: PLoS Negl Trop Dis. 2019 Jan 11;13(1):e0007075. doi: 10.1371/journal.pntd.0007075 (PMC6329495; doi:10.1371/journal.pntd.0007075)
Supplement: S1 Text — (DOCX) [file pntd.0007075.s002.docx]

**S1 Text. Questionnaire used for data collection**

**A. Socio-demographic characteristics:**

1. Completed age in years
   1. 18-24 b. 25-29 c. 30-34 d. 35-39 e. 40-44 f. 45 or above
2. Sex
   1. Female b. Male c. Others
3. Ethnicity
   1. Dalit b. Backward Janajati c. Higher Janajati

d. Other Backward Caste e. Minority Religious Society

f. Brahmin/Chhetri

1. Religion
   1. Hindu b. Muslim c. Christian d. Others
2. Residence/districts
   1. Dhanusha b. Parsa
3. Marital status
   1. Married b. Unmarried c. Widow/Widower d. Divorced e. Separated
4. Type of family:
   1. Nuclear b. Joint
5. Family affection:

Anybody in family affected by leprosy? A. Yes B. No

Anybody in close relationship affected by leprosy? A. Yes B. No

Anybody in neighbor affected by leprosy? A. Yes B. No

1. Occupation: What is your occupation?
   1. Farmer b. Laborer c. Business d. Service e. Housewife

f. Student g. Unemployed h. Others

1. What is the source of income?
   1. Agriculture
   2. Governmental Job
   3. Private Job
   4. Foreign Employment
   5. Business
   6. Other
2. How much does monthly income amount?
   1. ≤ 8000 NRS
   2. Between 8000 NRS to 12000 NRS
   3. Between 12000 NRS to 16000 NRS
   4. Between 16000 NRS to 20000 NRS
   5. 20000 NRS or above
3. Is that income generation enough to sustain living conditions?
   1. Yes
   2. No

**B. Knowledge about leprosy**

1. Have you heard about leprosy?

- - 1. Yes
    2. No

*If yes,* where did you get the information from (several selections possible)?

1. Hospital
2. Local health workers
3. TV/Newspaper/Radio
4. Friend or family
5. Other

2. Do you know what causes leprosy? □ Yes □ No

*If Yes,* Please choose one of these:

- 1. Bacteria or any micro-organism
  2. Curse by god
  3. Karma
  4. Unclean environment
  5. Eating too much cool food
  6. Is hereditary
  7. Due to bad blood
  8. Others

3. Do you think leprosy is very infectious?

- 1. Yes
  2. No

4. Do you know how leprosy is transmitted? □ Yes □ No

1. From animal
2. From mosquito
3. air or flatus of the patients
4. bathing in a river with leprosy patients
5. contaminated soil
6. sexual contact with leprosy patients
7. prolonged close contact with leprosy patients
8. sitting close to the leprosy patients
9. sharing personal items (toothbrush, towels, etc) with leprosy patients
10. Other: specify

5. Do you think leprosy can be treated?

1. Yes
2. No

If yes, what is its treatment?

- 1. Pharmaceutical drugs
  2. Medicinal herbs
  3. Religious rituals

7. Do you think leprosy is a severe disease?

1. Yes
2. No

7. Do you know the signs and symptoms of leprosy? *If Yes,*

1. Patches
2. Tingling
3. skin irritation or itchiness
4. Loss of sensation
5. Deformity
6. Ulcer
7. Don’t know

9. What is the first sign/symptom of leprosy?

- 1. Skin involvement
  2. Nerve involvement
  3. Skin and nerve involvement
  4. Deformity

10. Do you go to a doctor/hospital as soon as you get to know of yourself being leprosy affected?

- 1. yes
  2. no

*If no*, why you will not seek for medical help (several selections possible)?

a. Have to ask head of the family

b. Feel ashamed

c. Don’t know it was Leprosy

d. Await for self-cure

**C. Attitudes of community towards leprosy**

| **S.N.** | **Attitude towards leprosy** | **Yes** | **No** |
| --- | --- | --- | --- |
| 1 | Sit side by side with leprosy patient in a  public conveyance |  |  |
| 2 | Avoid a leprosy patient  For example, avoid having food or  activities with him/her |  |  |
| 3 | Share food with the patients |  |  |
| 4 | Marry to family members who have  history of leprosy |  |  |
| 5 | It is difficult for leprosy patient to get  Married |  |  |
| 6 | Work in the same environment with a  leprosy patient |  |  |
| 7 | Allow your children to playing with a  child of a leprosy patient |  |  |
| 8 | Feel ashamed to tell others if having  any leprosy patient in your family |  |  |
| 9 | Take food cooked by leprosy patients |  |  |
| 10 | Support them throughout the duration  of treatment of there is affected family  members |  |  |
| 11 | Participate on healthy education  regarding leprosy if any |  |  |
| 12 | Help leprosy patients financially if he or  she is unable to work |  |  |
| 13 | Share cloths or other materials with  cured leprosy patients |  |  |

**D. Explanatory Model Interview Catalogue (EMICc) stigma scale**

| **No.** |  | **Yes** | **Possibly** | **Uncertain** | **No** | **Score** |
| --- | --- | --- | --- | --- | --- | --- |
|  |  | **2** | **1** | **0** | **0** |  |
| 1. | Keep others from knowing leprosy status if possible |  |  |  |  |  |
| 2. | Think less of yourself due to leprosy affected individual in family |  |  |  |  |  |
| 3. | Leprosy has caused shame or embarrassment in community |  |  |  |  |  |
| 4. | Others think less of a person with leprosy |  |  |  |  |  |
| 5. | Adverse effect on others if they know someone’s status of leprosy |  |  |  |  |  |
| 6. | Others would avoid a person with leprosy |  |  |  |  |  |
| 7. | Others would refuse to visit home of leprosy affected individual |  |  |  |  |  |
| 8. | Other people think less of a family with leprosy patient |  |  |  |  |  |
| 9. | Causes problem for family if anyone in family has leprosy |  |  |  |  |  |
| 10. | Disclosure concern by family to share leprosy status to others |  |  |  |  |  |
| 11 | Leprosy would cause problem to get married |  |  |  |  |  |
| 12. | Leprosy would cause problem in an ongoing marriage |  |  |  |  |  |
| 13. | Leprosy would cause problem in marriage of relatives |  |  |  |  |  |
| 14. | It is difficult to find work/job for a leprosy affected individual |  |  |  |  |  |
| 15. | Others would dislike to buy foods from a leprosy affected individual |  |  |  |  |  |
